# A Light Deep Learning Algorithm for CT Diagnosis of COVID-19 Pneumonia

**DOI:** 10.3390/diagnostics12071527

**Published:** 2022-06-23

**Authors:** Adhvan Furtado, Carlos Alberto Campos da Purificação, Roberto Badaró, Erick Giovani Sperandio Nascimento

**Affiliations:** 1 Supercomputing Center SENAI CIMATEC, Av. Orlando Gomes, 1845, Piatã, Salvador 41560-010, Brazil; adhvan@fieb.org.br (A.F.); carlos.purificacao@fieb.org.br (C.A.C.d.P.); 2 Instituto SENAI de Inovação em Saúde, Av. Orlando Gomes, 1845, Piatã, Salvador 41560-010, Brazil; badaro@fieb.org.br

**Keywords:** deep learning, COVID-19, CT, screening test

## Abstract

A large number of reports present artificial intelligence (AI) algorithms, which support pneumonia detection caused by COVID-19 from chest CT (computed tomography) scans. Only a few studies provided access to the source code, which limits the analysis of the out-of-distribution generalization ability. This study presents Cimatec-CovNet-19, a new light 3D convolutional neural network inspired by the VGG16 architecture that supports COVID-19 identification from chest CT scans. We trained the algorithm with a dataset of 3000 CT Scans (1500 COVID-19-positive) with images from different parts of the world, enhanced with 3000 images obtained with data augmentation techniques. We introduced a novel pre-processing approach to perform a slice-wise selection based solely on the lung CT masks and an empirically chosen threshold for the very first slice. It required only 16 slices from a CT examination to identify COVID-19. The model achieved a recall of 0.88, specificity of 0.88, ROC-AUC of 0.95, PR-AUC of 0.95, and F1-score of 0.88 on a test set with 414 samples (207 COVID-19). These results support Cimatec-CovNet-19 as a good and light screening tool for COVID-19 patients. The whole code is freely available for the scientific community.

## 1. Introduction

COVID-19 still affects public health services. Until 17 June 2022, there have been 535,863,950 confirmed cases of COVID-19 and 6,314,972 deaths all over the world, reported to WHO [1]. Despite the declining curve of new cases throughout the world, it is paramount to identify suspicious cases, differentiate them from other respiratory diseases, and to define appropriate isolation and treatment strategies [2]. In healthcare units, mechanisms for screening and monitoring the evolution of the disease are essential. The “Gold Standard” for diagnosing a COVID-19 infection is a reverse transcription-polymerase chain reaction (RT-PCR) test. Although RT-PCR is a reliable test, it needs trained people to perform the nasopharyngeal swab collection and a specialized laboratory for analysis. Results can take a few hours or days, and there is a significant and not yet fully explained variation in the proportion of false-negative results [3,4]. There are many healthcare facilities, especially in developing countries, where mechanisms for patient assessment and management are essential and RT-PCR is not completely available.

The SARS-CoV-2 infection generates characteristic abnormalities in chest image examinations. Chest radiography and computed tomography (CT) scans are the most common methods to support the diagnosis of pneumonia in symptomatic patients [5]. These examinations have been widely used as part of the initial screening and in situations where the patient has strong respiratory symptoms [6]. Even with the appearance of new variants less aggressive to lungs, it is still necessary to detect and monitor COVID-19 pneumonia, as we do not know how the disease will evolve in the next years to come.

**Figure 1:**
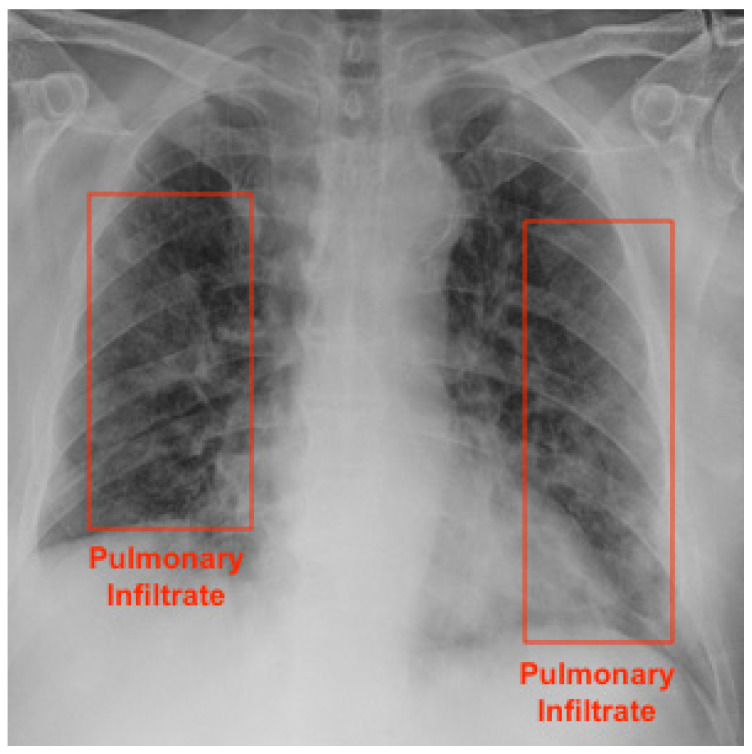
COVID-19 patient is male and 73 years old. Bounding box highlights infiltrates.

An X-ray machine is the most commonly available imaging tool for patients with respiratory complaints. It is especially useful to identify severe cases of COVID-19 patients, as there might not be any findings on exams in mild or early-stage patients [7]. It is a simple, fast, and safe examination procedure. AI algorithms can support the detection of pneumonia caused by COVID-19 in chest radiographs [8]. Figure 1 presents a COVID-19 patient’s radiography highlighting pulmonary infiltrates.

**Figure 2:**
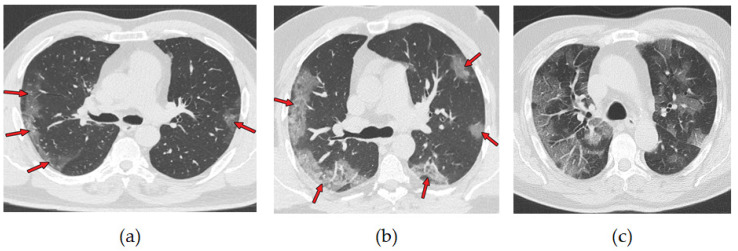
Axial nonenhanced chest CT images (lung window) in a 59-year-old man (**a**) and a 47-year-old man (**b**) show bilateral areas of ground-glass opacities (arrows) in a peripheral distribution; (**c**) shows bilateral ground-glass opacities and dilated segmental and subsegmental vessels, mainly on the right, in a 70-year-old man, each with positive RT-PCR test results for SARS-CoV-2. Adapted from [10].

A chest CT scan combines data from multiple X-rays taken from different angles, which produces a detailed image of the lungs. CT scans are more effective than chest X-ray in early stages of COVID-19 disease detection. They have been used as a tool to diagnose and monitor the progression of the disease [9]. More than 70% of chest CT scans in patients with RT-PCR test-proven COVID-19 cases report ground-glass opacities, vascular enlargement, bilateral abnormalities, lower lobe involvement, and posterior predilection [10]. Figure 2 illustrates those abnormalities. Studies by [11,12] confirm that patients with COVID-19 pneumonia have ground-glass opacities in the earlier stages of the disease and pulmonary consolidation in later stages. Eventually, a rounded morphology and a peripheral pulmonary distribution are observed. Those abnormalities are analogous to those observed in other coronavirus infections, such as SARS-CoV-1 and MERS-CoV [13].

Although typical images can help in the early screening of suspected cases, images of various viral pneumonias are similar and overlap with other infectious and inflammatory lung diseases. Therefore, it is not trivial for radiologists to distinguish COVID-19 pneumonia from other viral pneumonias. AI algorithms are a valuable tool to support this task. It is important to notice that the WHO and the American Society of Radiology do not recommend the use of radiology images as the principal diagnostic method for COVID-19 [14,15,16].

The perspective of using deep learning algorithms as a fast and widely available alternative for the diagnosis of COVID-19 by RT-PCR has expanded the quantity and quality of research in this area. A research done on 1 May 2022 for articles with the words: “Deep Learning” and “CT” and “COVID-19” and “Diagnosis” in the abstract resulted in 287 findings in the *PubMed* database, being 52 in the MDPI repository. Despite the availability of studies, there are strong obstacles for the regular application of the proposed algorithms in clinical practice. A study by [17] systematically reviewed publications of machine learning models for the diagnosis or prognosis of COVID-19 from X-ray or CT images, concluding that all identified models had methodological flaws and/or underlying biases preventing their use in clinical practice. A review by [18] identified that most of the studies have utilized small datasets and lacked comparative analysis with other existing research, and the codes and data were not available. In our review, we also identified fundamental problems that limit the adoption of algorithms in healthcare centers. There is limited access to the complete source code, train, and test data. Thus, it is not possible to replicate the results and to evaluate the AI algorithm on different data sets. Most of the studies used a limited number of images from local sources or used only well-known public databases, and therefore, their models were not stressed enough to generalize properly to other phenotypes and geographic regions contexts. For instance, we only identified a few publications that used chest CT images from Brazilian hospitals. The work by [19] used data from 130 patients from two hospitals in Rio de Janeiro and one in Porto, Portugal, to develop an algorithm to identify and quantify the extent of lung involvement in patients with COVID-19 pneumonia. The study by [20] developed an algorithm for segmenting COVID lesions on CT using a base of 40 patients from a hospital in Rio de Janeiro. Both studies used small databases. In this work, we avoided repeating the most common flaws identified in the available studies and sought to advance the knowledge necessary to support the use of such algorithms in clinical practice, preparing it for use in a hospital in Brazil, a country with resources constraints to combat COVID-19. Table 1 categorizes the mapped problems and solutions developed in this work.

A review study by [21] highlighted the widespread use of convolutional neural networks for extracting relevant features from CT scans and noted that most classification models for COVID-19 use pre-trained networks. Another extensive review done by [22] showed that many 2D and 3D models were used to support the identification of pneumonia, mainly based on Inception, VGG, and ResNet architectures.

The work of [23,24] used 2D networks to analyze each CT slice image individually and adopted voting methods to classify the patient outcome. Another popular approach using 2D networks was to generate embedding feature vectors for every image, pool them to a single global feature vector, and use fully-connection layers for classification [25,26]. Some studies used 3D CNN networks, where a subset or all the available CT slice images per examinations were used as input [27,28]. Most of the 3D CNN algorithms used a fixed number of images from CT examinations as input because using all available images can be very memory-consuming. The work by [29] studied and compared various deep learning techniques applied to both chest radiographs and CT scans images for the detection of COVID-19 and validated VGG16 and ResNet50 as good architectures for classification. In order to develop a new model for the COVID-19 diagnosis, the study by [30] tested multiple architectures: DenseNet-169, VGG-16, ResNet-50, InceptionV3, and VGG-19. The VGG-19 proved to be superior with an accuracy of 94.52% when compared to all other deep learning models. The similarity of COVID-19-generated pulmonary lesions with the ones generated by other respiratory diseases reinforces the necessity of the algorithm to have an excellent feature extraction ability. A study by [31] proposed the use of a bag of deep visual words (BoDVW) on the VGG-16 architecture. The method removes the feature-map normalization step and adds a deep feature normalization step on the raw feature maps, preserving the semantics of each feature map that might have importance in differentiating COVID-19 from other forms of pneumonia on radiographies. This method was improved by including a multi-scale BoDVW [32] and an attention module to capture the spatial relationship between the regions of interest in CXR images [33].

In our work, we decided to adapt the VGG architecture for a 3D CNN. The input is a set of slices of a patient’s CT. The objective is to preserve the embedded information of the CT examination on the frame stack, thus mimicking the behavior of a radiologist’s analysis. We used a fixed set of 16 slices per CT scan examination to reduce hardware consumption and avoid lack of memory problems. We developed a novel pre-processing technique to choose and prepare the best slices for training and validation.

There are many regions in Brazil and in the world that do not have access to RT-PCR exams in the quantity and time needed or specialized physicians. In these cases, alternatives that facilitate the diagnosis of COVID-19 are very important. In this paper, we present Cimatec-CovNet-19, a fast, VGG-based CNN algorithm for COVID-19 diagnosis in chest CT scans. We developed our system on a set of 3000 chest CT scans, from which 734 examinations were from Brazilian hospitals. This study confirms the hypothesis that AI systems are able to correctly classify COVID-19 and non-COVID-19 classes from CT scans. We evaluated and compared the performance of the algorithm with data from geographically distributed datasets and data from a Brazilian hospital. The main innovations of this study are:

- Proposing a novel 3D VGG-based CNN architecture for accurately diagnosing COVID-19 on chest CT scans. The 3D network is able to identify correlations between adjacent slices, while 2D networks are limited to intra-slice spatial voxel information.
- Introducing a novel pre-processing technique, which reduces the number of slices required for training the algorithm: Processing fewer slices demands less computational power, prevents communications bottlenecks, and reduces time and cost constraints. Since the model only requires 16 slices per CT examination, it is also well-suited for a large number of CT machines.
- Evaluating the algorithm’s diagnosis performance in both geographically distributed and Brazilian datasets: Brazil has more than 300,000,000 inhabitants. It was one of the worst-affected countries in the world by the COVID-19 pandemic. Despite that fact, there are few studies with data from this country. It was important to include images from Brazilian hospitals and confirm the algorithm’s ability to generalize well for this phenotype. We plan to test the algorithm in a controlled environment in a Brazilian hospital in the near future.
- Disposing the algorithm as an open software for public use and future enhancements: This guarantees reproducibility.

## 2. Materials and Methods

### 2.1. Dataset Preparation

In the retrospective study, we gathered 5787 CT scans from nine different datasets sources. We used seven public datasets containing CT scans from all over the world: *Medical Segmentation Decathlon, LNDb, LCTSC, MOSMEDDATA, COVID-19 CT Lung and Infection Segmentation, COVID-19 CT Segmentation Dataset, BIMCV-COVID19,* and two private datasets from Brazilian hospitals: *HCUSP* and *HSI*. We included in this study only images in the axial plane and from patients with a diagnosis issued by a radiologist from well-known hospitals. All patient information was already anonymized in the data source. The ground truth for a positive COVID-19 outcome was a positive RT-PCR test associated with the CT-scan examination. We performed a visual inspection of the central slice in each of the 5787 CT scans and manually discarded all data that were in sagittal or coronal planes, had low-quality resolution, or were masks of CT scans instead of the CT scan itself. Altogether, this procedure removed 1108 samples. Table 2 presents the complete list of databases used in this work.

Considering a variety of CT scanners available worldwide, it would be natural to expect that the source datasets had different number of slices and resolutions, which, in fact, happened. Additionally, the data were unbalanced regarding the presence of COVID-19-positive CT scans. The demographic information from the patients was not consistent and thus not used in this work. From the remaining 4679 CT scans, we prepared a random, balanced subset with 3000 samples (1500 COVID-19, 1500 non-COVID-19), which were then split into training and validation sets.

### 2.2. Dataset Description

- *Medical Segmentation Decathlon: The Medical Segmentation Decathlon* is a collection of annotated medical image datasets for the development and evaluation of segmentation algorithms. The lung dataset has 96 preoperative thin-section CT scans performed without use of contrast and from patients with non-small cell lung cancer from Stanford University (Palo Alto, CA, USA) publicly available through TCIA [34].
- *LNDb*: This dataset contains 294 CT scans collected retrospectively at the Centro Hospitalar e Universitário de São João (CHUSJ) in Porto, Portugal, between 2016 and 2018. All data were acquired under approval from the CHUSJ Ethical Committee and was anonymized. Among the 294 patients scanned, 164 (55.8%) were male. The average age was 66, and the minimum and maximum ages were 19 and 98, respectively [35].
- *LCTSC*: This dataset was provided in association with a challenge competition and related conference session conducted at the American Association of Physicists in Medicine 2017 Annual Meeting. There are CT scans of 60 patients undergoing treatment simulation for thoracic radiotherapy from three institutions: MD Anderson Cancer Center, Memorial Sloan-Kettering Cancer Center, and the MAASTRO clinic. Each institution provided CT scans from 20 patients, including mean intensity projection (4D CT), exhale phase (4D CT), or free-breathing CT scans depending on their clinical practice. All CT scans covered the entire thoracic region with a 50 cm field of view and slice spacing of 1 mm, 2.5 mm, or 3 mm [36].
- *MOSMEDDATA*: This dataset contains 1110 anonymized lung CT scans obtained between 1 March 2020 and 25 April 2020 from public medical hospitals in Moscow, Russia. Among the patients scanned, there were 42% males, 56% females, and 2% other/unknown with ages from 18 to 97 years, with an average of 47 years. They were distributed according to a classification table of the severity of lung tissue abnormalities with COVID-19 and routing rules. There were five categories ranging from CT-0, zero, not consistent with pneumonia (including COVID-19) up to CT-4, severe, with diffuse ground glass opacities, with consolidations and reticular changes, and pulmonary parenchymal involvement ≥ 75%. The number of cases by category was: CT-0, 254 (22.8%); CT-1, 684 (61.6%); CT-2, 125 (11.3%); CT-3, 45 (4.1%); and CT-4, 2 (0.2%) [37].
- *HC USP*: The data were obtained through a collaboration between SENAI CIMATEC and the Medical School of the University of São Paulo (HC USP). Altogether, we obtained 439 COVID-19-positive exams and 506 COVID-19-negative exams.
- *HSI*: The data were acquired from a partnership between SENAI CIMATEC and the Santa Izabel Hospital (HSI). This database has 1294 COVID-19-positive exams and 512 COVID-19-negative exams.
- *COVID-19 CT Lung and Infection Segmentation*: This dataset contains 20 labeled COVID-19 lung-infection CT scans collected from the Coronacases Initiative and Radiopaedia, which can be freely downloaded with CC BY-NC-SA license. The proportion of infections in the lungs ranges from 0.01% to 59%. The left lung, right lung, and infection segmentation were firstly delineated by junior annotators (1 to 5 years of experience), then refined by two radiologists with 5 to 10 years of experience. All the annotations were verified and refined by a senior radiologist (>10 years of experience) [38].
- *COVID-19 CT Segmentation Dataset*: This dataset contains 100 axial CT images from more than 40 patients with COVID-19 converted from openly accessible JPG images provided by the Società Italiana di Radiologia Medica e Inteventistica. The images were segmented by a radiologist using three labels: ground glass, consolidation, and pleural effusion [39].
- *BIMCV-COVID19*: A large dataset from the Valencian Region Medical ImageBank (BIMCV) containing chest X-ray images CXR (CR, DX) and computed tomography (CT) imaging of COVID-19+ patients along with their radiological findings and locations, pathologies, radiological reports (in Spanish), DICOM metadata, polymerase chain reaction (PCR), immunoglobulin G (IgG), and Immunoglobulin M (IgM) diagnostic antibody tests was also used. This database includes 1380 CX, 885 DX, and 163 CT studies from 1311 COVID-19 patients [40].

### 2.3. Slice-Wise Selection

In order to normalize the input resolution, we used the Clara Training framework, part of the Clara Image software suite, to resample all DICOM and NIfTI data to a voxel spacing resolution of 1 × 1 × 1 mm NIfTI format. Clara is an application framework optimized for healthcare and life sciences developers. It contains software development kits, full-stack GPU-accelerated libraries, and pre-tested reference applications [41]. We also used the Clara framework to obtain lung masks from each chest CT scan. We used the clara_train_covid19_ct_lung_seg model, a voxel-wise binary classification for lung region segmentation. Each voxel is predicted as either foreground (lung) or background. The output is a binary mask, where the lung is assigned 1, and the background is assigned 0. We noticed that the sum of pixels in the lung masks grows in a Gaussian-like pattern from the first to the last slice, peaking around the central slice. Using this information, we did a slice-wise selection in order to collect data from different areas of the lung. After experimenting with 64, 32 and 16 slices, the results did not have any significant statistical differences, so we used 16 slices from each CT scan in order to save computational resources. The slice-wise selection was performed according to the following expression:

slice_i_ = *F* + *G* × i, with i in [0, 1, 2, …, 15], (1)

where *F* is the first slice in the mask whose sum of pixels is greater than 1000, and *G* is the step size given by:

*G* = ⌈(*µ* − *F*)/8)⌉, (2)

with *µ* being the central slice.

**Figure 3:**
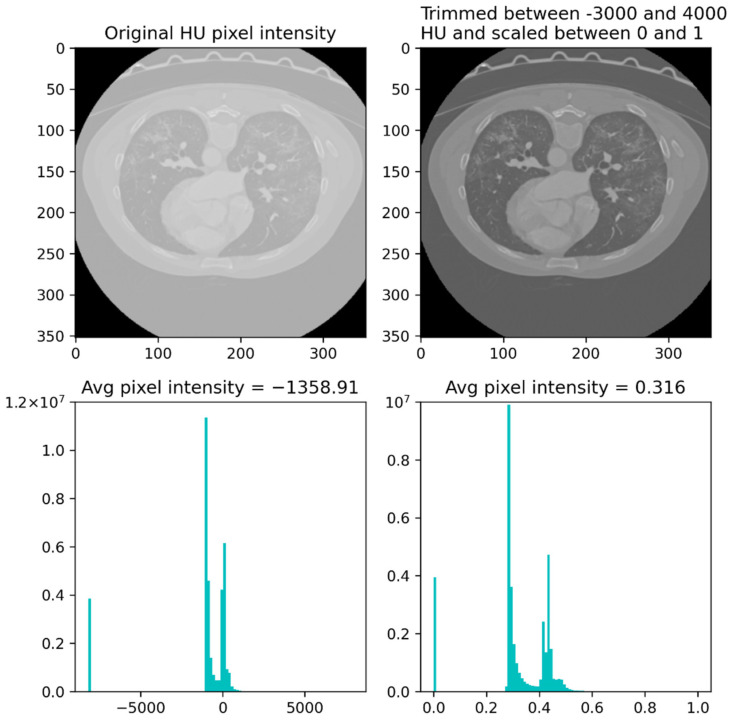
Example of a single slice before and after the pre-processing routine.

Before executing the described slice-wise selection, the CT scans were trimmed between –3000 and 4000 Housefield units (HU) and scaled between 0 and 1. We reshaped the 16 slices chosen from each CT scan to a 512 × 512 × 16 × 1 format. Figure 3 depicts a single slice from an exam both before and after being pre-processed.

### 2.4. Algorithm Architecture

Cimatec-CovNet-19 has an architecture inspired by the VGG-16 neural network. The VGG-16 was developed in 2014 and is one of the best CNN architectures to deal with 2D large-scale image recognition tasks. The image passes through a stack of convolutional layers with very small receptive fields (3 × 3 kernels), which is the smallest size possible to capture pixel position notions (left/right, up/down, center). The spatial resolution is preserved with paddings. After some of the convolutional layers, there are max pooling layers (2 × 2 window, stride 2) to guarantee spatial pooling. The stack of convolutional layers is followed by three fully connected (FC) layers. The last layer is a softmax layer, which is a function to represent the network output as a categorical distribution [42].

**Figure 4:**
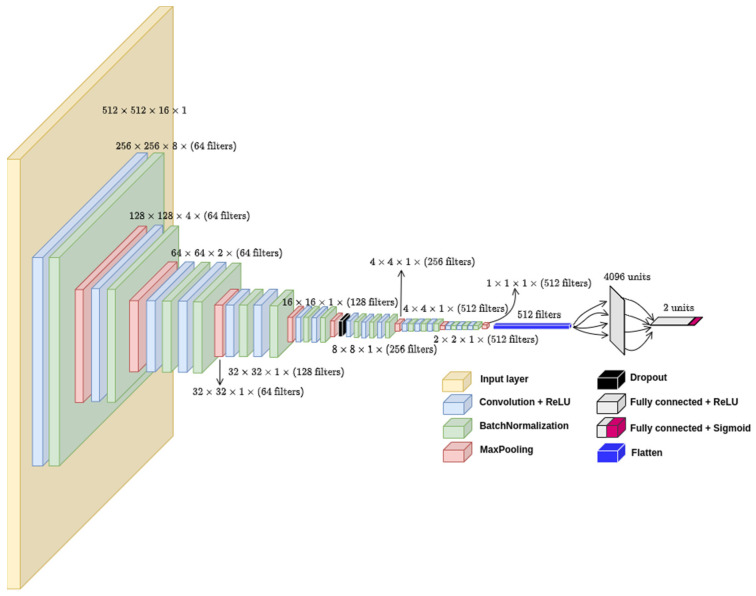
3D-CNN model architecture developed on top of the standard VGG-16 2D model.

In our model, there are 17 convolutional layers split into 5 convolutional blocks with different filter sizes, as can be seen in more detail in Figure 4.

The model takes CT slices as input and combines the features extracted from the slices in a sequence of convolutions and pooling operations. The number of input slices can vary. Typically, it can be 64, 32, or 16 slices. It requires an analysis and validation of the approach to select the lowest number of slices without losing accuracy, which will be presented in the following section.

There are more pooling layers in the two initial convolutional blocks than in the final ones. We chose this approach to reduce the tensors size and fit them in the available GPU memory. We also added batch normalization layers after every convolutional layer and a single dropout layer with a 0.5 dropout rate to enhance the training performance and prevent overfitting. The final feature map runs through two FC layers, the first with 4096 neurons and the second being the output layer with a sigmoid activation function to generate a binary output, namely COVID-19 or non-COVID-19. All hidden layers are built with the rectified linear unit (ReLU) [43] activation function. The model had 47.3 million parameters, was trained in a computing node with four NVIDIA GPUs V100 32 GB SXM2, and took 9313 s to train 56 epochs.

### 2.5. Model Training

We randomly initiated the weights and trained the neural network with a batch size of 16 using the Adamax optimizer and learning rate of 10^−3^. We used early stopping with a patience of seven epochs based on the validation loss. During model development, 2000 samples were used for training and 1000 samples for validation as observed in Table 3.

In order to fine-tune the CNN architecture, we started the experiment with a different number of convolutional and pooling layers, following the VGG-16 pattern (increasing the filter size as the layers went deeper). Then, we tried different number of neurons in the FC layers and a sequence of three FC layers. Finally, we tried different regularization techniques:

- Batch normalization layer in different positions after the convolutional layers,
- Dropout layers in different positions and different dropout rates,
- L2 regularization in different layers, resulting in regularizations to the fourth, eleventh, and fourteenth convolutional layers and to the penultimate FC layer.

All the experiments were performed with the keras tuner API [44], which is an easy-to-use, scalable, hyperparameter optimization framework. We performed the hyperparameter search with the built-in hyperband optimization algorithm [45].

We used two datasets for model assessment: (1) data from Medical School of the University of São Paulo and (2) data randomly taken from the full dataset. Both test sets were balanced (50% for each class: COVID-19, non-COVID-19). We reached a plateau for model assessment after experimenting with several different hyperparameters settings and model architectures.

In order to evaluate the model variability in different portions of the data, we used a stratified 10-fold cross-validation on the 3000 samples. Finally, we combined the training and validation datasets into a single training dataset and added data augmentation to each of the 3000 examples, bringing the total number of samples in the training dataset to 6000, as observed in Table 4.

Five different data augmentation techniques were tried: vertical and horizontal flip, changing brightness and contrast, shear, zoom-in and zoom-out, and small rotations. For each technique, we trained the model with a pair-wise combination of the 3000 original images with 3000 augmented images. Finally, we combined all augmented images with the original images and found that augmented rotated images showed the best results. In this technique, every image suffered small rotations. The algorithm randomly rotates the images with one of the angles in the set (−15, −10, 10, 15). For the final training, there were neither validation data nor automatic early stoppage. We defined the number of epochs to train the algorithm as 56. It was the same number of epochs achieved for the best model weights reached during model development.

## 3. Results

**Figure 5:**
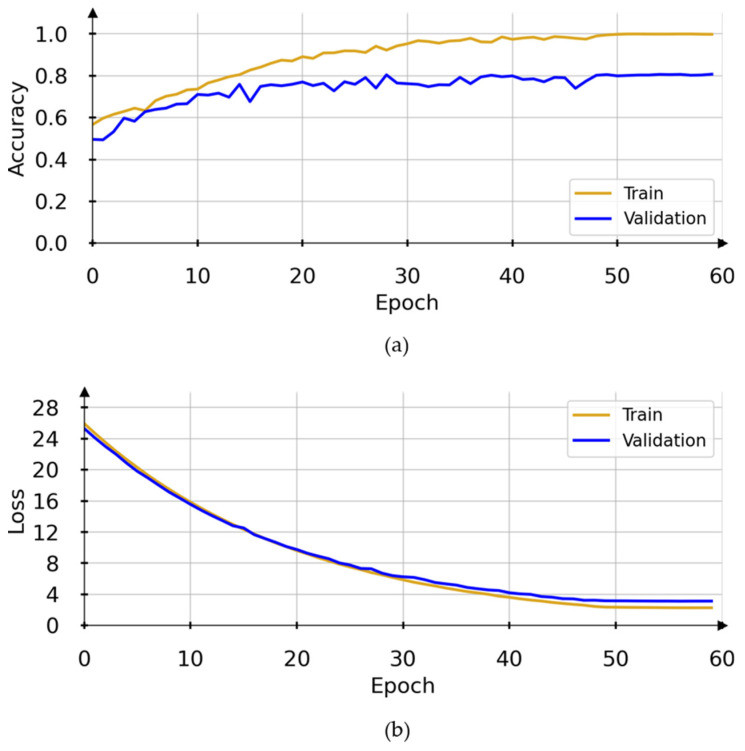
Model performance achieved during the development. In (**a**), training and validation accuracy. In (**b**), training and validation loss.

After trying different hyperparameters setups throughout model development, we achieved the results presented on Figure 5. Notice that the validation curves reach an accuracy plateau around 0.80 by the 50th epoch. The model weights stabilize, and the accuracy for both training and validation data show little changes. The loss for the training and validation sets also stabilizes around epoch 50.

**Figure 6:**
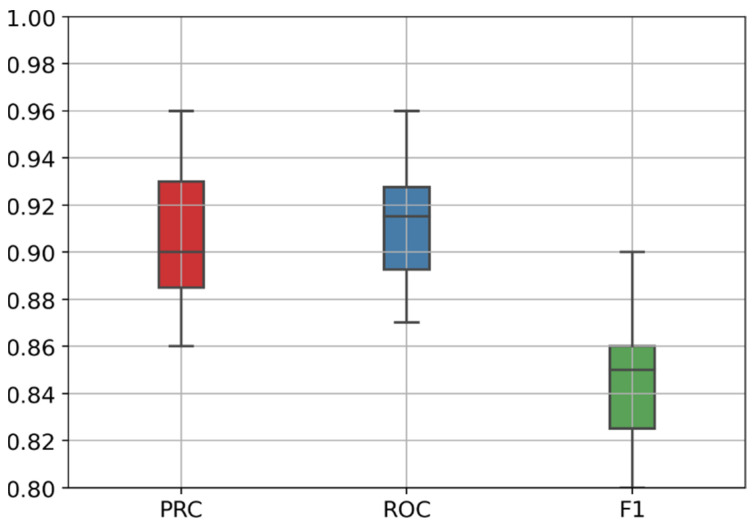
Boxplot of the stratified 10-fold cross-validation results showing PR-AUC, ROC-AUC, and F1-score ranges.

Figure 6 presents the boxplot of the stratified 10-fold cross-validation results, and Table 5 presents the evaluation results for each validation fold in more detail. We can observe that the PR-AUC varies from 0.86 to 0.96, the ROC-AUC varies from 0.87 to 0.96, and the F1-score varies from 0.80 to 0.90. Those results represent a good overall performance when compared to several recent related works [21,46,47].

**Figure 7:**
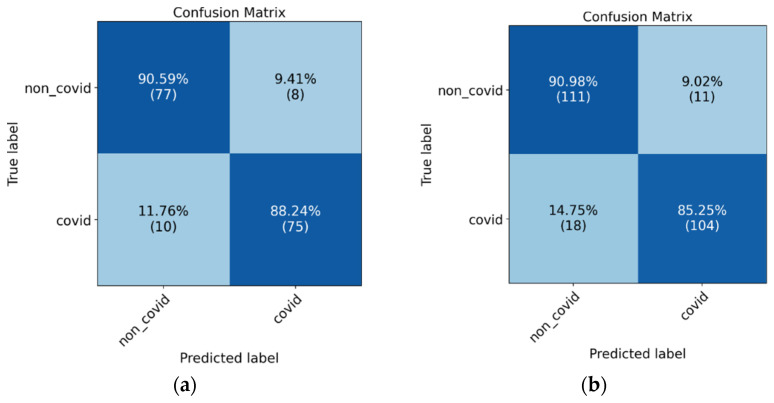
Confusion matrix results for model evaluation on the test datasets, (**a**) test dataset 1, and (**b**) test dataset 2.

**Figure 8:**
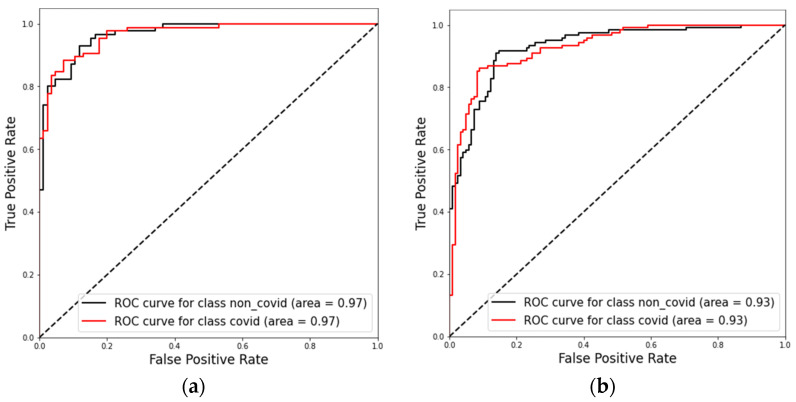
ROC curves obtained for model evaluation on the test dataset 1 (**a**) and on the test dataset 2 (**b**).

**Figure 9:**
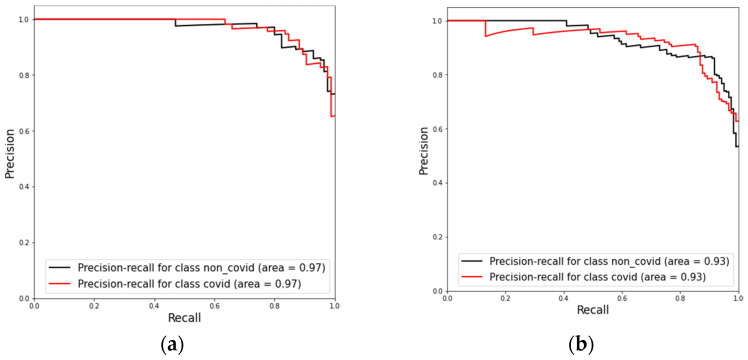
Precision-recall curves for model evaluation on both test datasets. (**a**) Results for test dataset 1 and (**b**) results for test dataset 2.

The confusion matrices in Figure 7 the ROC-AUC in Figure 8 and PR-AUC in Figure 9 show the model performance in both test datasets. For test dataset 1, the model assessment shows a recall of 88.51% (95% CI, 79.88% to 94.35%), specificity of 90.36% (95% CI, 81.89% to 95.75%), accuracy of 89.41% (95% CI, 83.78% to 93.60%), and ROC-AUC and PR-AUC of 97%. Test dataset 2 shows a recall of 85.25% (95% CI, 77.69% to 91.02%), specificity 90.98% (95% CI, 84.44% to 95.41%), accuracy of 88.11% (95% CI, 83.38% to 91.89%), and ROC-AUC and PR-AUC of 93%.

**Figure 10:**
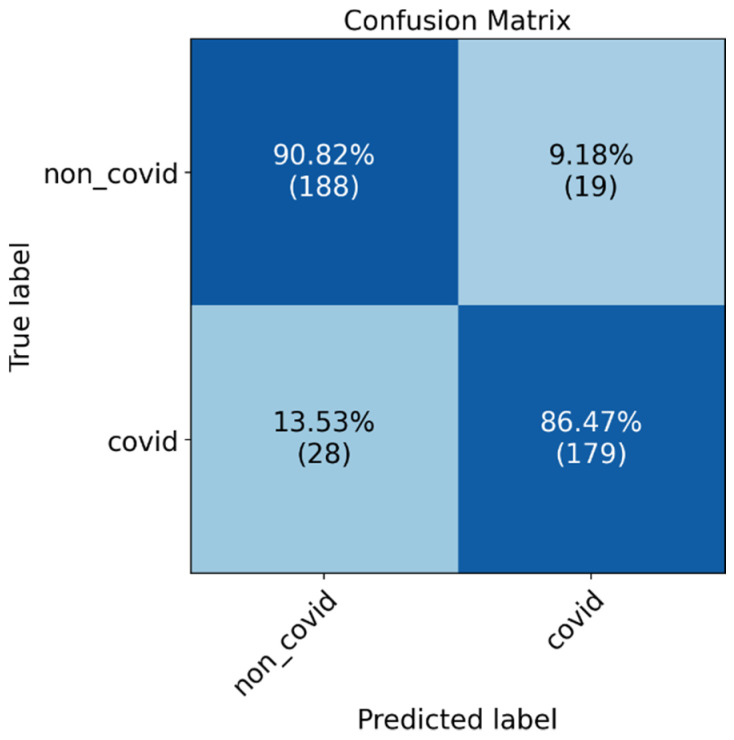
Confusion matrix for model evaluation on the combined test dataset.

**Figure 11:**
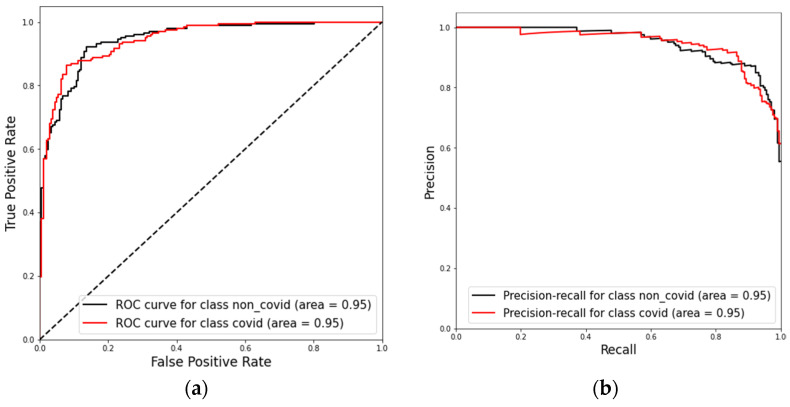
Present the ROC-AUC (**a**) and PR-AUC (**b**) results for model evaluations on the combined test dataset.

Finally, we present the results with the combined datasets (test dataset 1 and test dataset 2) in Figure 10 and Figure 11 as an overall performance assessment. The model assessment shows a recall of 88% (95% CI, 79.88% to 94.35%), specificity of 88% (95% CI, 81.89% to 95.75%), and accuracy of 89% (95% CI, 83.78% to 93.60%). We can see a ROC AUC and PR AUC of 95% for the combined test dataset. The model’s performance in both dataset and in the combined set confirms its ability to generalize well for new data.

## 4. Discussion

In the lack of a specialized radiologist, AI models may support the identification of COVID-19 pneumonia characteristics in CT scans. With this objective in mind, we developed the Cimatec-CovNet-19 neural network and evaluated its performance using two test datasets: one being a subset of a global public dataset and the other a set of 170 patients served by a hospital in São Paulo. Generalization for different datasets is a known problem in AI applied to medical images [48]. We did not observe major differences in the algorithm performance over the two tests datasets, which suggests that the algorithm generalizes well.

One limitation of this study is the use of a diverse public dataset, which lacks demographic information to train the algorithm. Those datasets might contain unknown biases and contaminate the model.

The importance of CT scans examinations to evaluate suspected COVID-19 patients and support the management of known patients is evident. The ROC-AUC and PR-AUC showed in this study validated that Cimatec-CovNet-19 is a good screening tool for COVID-19 pneumonia from CT scans. The algorithm has a new approach for processing the images, requiring the use of fewer slices per examination and thus reducing training and inference times. This is important, especially for centers with low computing resources. The code is open for further enhancement. We encourage future works to compare this algorithm with other publicly available algorithms and explore its use in clinical practice in a controlled environment. In the near future, we plan to test Cimatec_CovNet-19 in a hospital in Brazil.

The methodology used to build and test the algorithm and the developed model can quickly be adapted and applied to other lung infections in new potential pandemics.

## Figures and Tables

**Table 1:** Assessment of the main problems found in the literature review.

Category	Problem	Solution
Dataset	Few images for training the algorithm	Algorithm trained with 3000 chest CT examinations
Dataset	Data collected from only one geographical region	Data collected from a set of curated international public databases summed up with images from 2 Brazilian hospitals
Dataset	Poor image bank quality: non-standard scans, too many images of children, or excess of data from China patients	Attention to the selection of the best public bases; automatic and visual cleaning.
Methodology	Use of unbalanced datasets	Attention to balancing the COVID-19, non-COVID, and normal categories prior to training and testing.
Methodology	Lack of statistical rigor or bias	We used the CLAIM [10] checklist for AI in medical imaging. Available as a supplement.
Transparency	Non-replicable projects.	The whole code is open.

**Table 2:** CT scans databases used in this study.

Dataset ID	Dataset Name	Public (Y/N)	Number of CT Scans after Data Cleaning	Avg Number of Slices per CT Scan	Number of CT Scans Positive for COVID-19	Training/Val Dataset	Test Dataset 1	Test Dataset 2
i	*Medical Segmentation* *Decathlon*	Y	94	279	0	50	0	9
ii	*LNDb*	Y	139	322	0	78	0	13
iii	*LCTSC*	Y	94	279	0	25	0	3
iv	*MOSMEDDATA*	Y	1105	42	1105	449	0	56
v	*HCUSP*	N	935	337	431	384	170	51
vi	*HSI*	N	1806	308	1294	766	0	79
vii	*COVID-19 CT Lung* *and Infection Segmentation*	Y	10	176	10	8	0	1
viii	*COVID-19 CT* *Segmentation Dataset*	Y	10	280	10	3	0	1
ix	*BIMCV-COVID19*	Y	486	288	308	222	0	31

**Table 3:** Dataset split during model development.

	Training	Validation	Test Dataset 1	Test Dataset 2
COVID-19	1000	500	85	122
Non-COVID-19	1000	500	85	122
Total	2000	1000	170	244

**Table 4:** Final training dataset with data augmentation.

	Original	Augmented	Test Dataset 1	Test Dataset 2
**COVID-19**	1500	1500	85	122
**Non-COVID-19**	1500	1500	85	122
**Total**	3000	3000	170	244

**Table 5:** Evaluation results for each validation fold.

Fold	PR-AUC	ROC-AUC	F1-Score
0	0.94	0.93	0.86
1	0.86	0.87	0.80
2	0.88	0.89	0.82
3	0.90	0.91	0.84
4	0.94	0.93	0.86
5	0.90	0.92	0.87
6	0.88	0.89	0.84
7	0.96	0.96	0.90
8	0.90	0.92	0.86
9	0.90	0.90	0.82
**Average**	0.906	0.912	0.847
**Std**	0.031	0.026	0.029

## Data Availability

The model’s source code is freely available on the research group GitHub page at https://github.com/CRIA-CIMATEC/COVID-19 (accessed on 1 May 2022).
